# A Discussion on Abortion in the Chicago Medical Legal Society

**Published:** 1891-01

**Authors:** 


For Daniel's Texas Medical Journal.

### A DISCUSSION ON ABORTION, IN THE CHICAGO MEDICO-LEGAL SOCIETY.

[The discussion refers to an anonymous article on abortion which appeared in the N. Y. Medico-Legal Journal, and which article rather excused abortions and abortionists.—ED.]

JUDGE O. M. HORTON:—My attention was called by your president to the article to which reference has been made, and last night I read it. I have no prepared address, but as I read the article I jotted along the margin some points that occurred to me. The reader of the paper, and also your president, expressed the opinion that this article was written by a lawyer. I should want to call for the evidence on that question; I see nothing to indicate that it is a lawyer any more than a physician who wrote it, and I propose to defend the legal profession until you bring better evidence than the suspicion of those members of the medical profession who seemingly want to get rid of a gentleman who would write such an article. After several pages he commences with a sub-heading which he calls the argument, and his first expression is that abortion is nowhere forbidden in the Bible. The word abortion, I fancy, does not occur in the Bible, and if the writer must depend upon a word that exactly tallies with the word he has seen fit to select as the topic of his article, then the Bible does not prohibit it. But among the very first utterances we read, "Be fruitful and multiply and replenish the earth." I apprehend that there is something in that that means we shall not destroy human life; I fancy that it needs no argument to tell us that it is a prohibition of the destruction of human life. You gentlemen of the medical profession can determine where human life begins better than I can. Again, we find, "Thou shalt not kill," If it be crime to kill a child an hour after birth what is it to kill it an hour before? If wrong to kill immediately after, is it not wrong to kill immediately before? And if wrong to kill immediately, then how long before birth does it cease to be wrong and become right? Now you have charged this anonymous article upon the legal profession. Not very long since a gentleman of your profession, Mr. President, under oath in a court of justice, when the court propounded to him the question as to whether the defendant knew the difference between right and wrong, answered that that old theory was exploded, that there was no such thing as a difference between right and wrong. I do not have to draw upon my imagination to tell what profession that gentleman belongs to.

You must pardon me for being a little rambling, but I have nothing but check marks on the margin of this article. He says here, in substance, that all English-speaking peoples, from a time so far back that the memory of man runneth not to the contrary, have regarded this as a crime and legislated against it. Now I submit to you that in almost, if not in every topic that you ever consider, the consensus of civilized humanity is a pretty safe thing to rely upon. I am not a woman's rights man, Mr. Chairman, in the ordinary sense of that term, but I protest against any anonymous writer charging home upon that sex, that stands beyond where you and I will ever attain in social relations, in delicacy of feeling, in refinement of thought—in these words, that "it is almost the universal sentiment of womankind that abortion is proper." I must believe, sir, that it is a libel upon the sex. Upon a topic of this kind I do not know just how to consider some of the authorities the writer refers to. I remember reading somewhere a very elaborate article with well considered, finely rounded sentences, on "How to rear children," and when carefully examined, it seemed to have come from the mind of an experienced woman, but further investigation demonstrated that it was written by an ancient maiden lady. Now what she knew about rearing children is more than I can tell. I remember that not a thousand miles from this city there was a meeting called, a religious meeting, a mother's meeting, and it was held by seven worthy, excellent, virtuous, pure minded women, four of whom were childless wives, and three old maids. I imagine, sir, as Gail Hamilton is quoted as authority upon this subject of abortion that it is not a very reliable authority, although she is a most charming and instructive writer. Twice does the writer repeat this, to my mind, slander upon womankind, in stating that it is the universal sentiment of woman that there should be no laws against abortion. He asks us, "Why not have the courage to meet the issue fairly and abolish all our laws on the subject, as did the laws of Moses, and leave the question to the moral sense and training of our women?" If it is morally wrong, why should it not be legally wrong? If it is morally a crime—if I may use that expression—why should not the law pronounce it a crime? Is this apologist prepared to say that abortion is morally right? I fancy not. The topic, as has been indicated, is divided, so that the second general branch is abortion in a criminal aspect for the unmarried. This may be the sentence from which you infer that he is a lawyer. He says, "Is there a lawyer with human blood in his veins who would condemn the fair young daughter of his best friend for the salvation of her honor *by this means?* I say, yes sir! There is one, and I defy you sir, to go into the legal profession and find the lawyer, who stands high in his profession, that will not say, here's another. And I believe, you may, with the same confidence, say, that you defy any one to go into your profession and find any man—who from the moral standpoint he occupies will not produce abortion—who will not say here is a doctor who believes the same. But he comes to the doctors, also. "Is there a physician," he says, "who has ever been a father, whose heart would be against such a woman's act to save herself from degradation, despair, death?" This argument is based upon the idea that there is no other way to avoid the social ostracism. If there is no other way, then meet it as becomes an honest individual. There are, perhaps, other ways besides publishing it, but he confines himself to that.

If it is morally wrong, then society is wrong when it socially ostracises the unfortunate. If it should not be declared to be legally wrong, if it is not morally wrong, then society is wrong when it condemns the person. I am not competent, Mr. President, to say much about Malhus, he is out of my line.

It seems to me that the whole air of this article, the spirit running through it, is that of a sort of infidel free-lovism; I hardly know how to define it. It is possible that there is something of that in the man's mind, and that he has not the boldness to stand out, as he asks others to stand, and say what he himself believes —in indiscriminate free love. Is that what he means; is that why he has written such an article? That article is an excellent one in favor of the old free love system of those sisters in New York—Tennessee Claflin and her sister. This article would be excellent in support of their theories; if their theories are wrong, this article is wrong. Possibly he is one of their converts, a sort of a decayed old doctor who has nothing else to do but prepare such articles, and he has not got the teachings of Tennessee and her sister out his veins yet. Possibly—I will not assert it—but possibly he is a sort of living advertisement for the vendors of abortion appliances. I do not know but they could well afford to pay him, it is a good article for them, and if his theory is adopted, their trade must be very largely advanced. You, sir, or any who send their daughters to public or to private schools, where catalogues of the names of students are published, will receive from time to time the vilest advertisements of the wares such men employ in their nefarious traffic. Is that one of the things we want to uphold? I think not. When the time comes that any community will justify the pollution of the morals of young women by throwing into their hands that vile advertising trash, that will be the knell of the republic, for no people that would thus willingly corrupt the morals of their young ladies are ever capable of self-government, nor can they maintain it long.

I have come to the last of my check marks on the margin of this thing; I tried to read it a second time, but I thought I had enough in reading it the first time to make a fair sort of talk upon it, and I submit that in my judgment it is the duty of this society, it is the duty of the medical profession to allow their voices to be heard, to be outspoken, fair, honest, manly in denunciation of that kind of teaching. And that a journal, purporting to be a medico-legal journal, ought never to publich such an article, let it emanate from whatever source it may.

Dr. Sarah Hackett Stevenson:—Mr. President, I feel entirely unprepared to discuss the subject. The article in question is an entirely new one to me, as I never heard of it before coming to this room. That an article of that nature should be found in a journal which has any influence or standing in the medical or legal profession, is, I think, its great danger. If it had been published elsewhere, it seems to me, it would be dignified on our part to pass it by without comment, but as it is found in such a journal, it certainly merits our rebuke. It was not necessary, Mr. President, for me to go to a medical college to learn that the destruction of life is murder and that murder is a crime. I learned that with my early education, and what has guided me is not the law of the land but an enlightened conscience, an educated conscience, and I feel very deeply that the man or woman who does not enter the medical profession with such a conscience. had better seek some other employment, it is not the place for him or her. I was very glad, indeed, that Judge Horton came to the rescue of my sex—that the enlightened women of this country are not in sympathy with the spirit of that article. As a physician my experience has been that the women who have asked for such a service, have done it through ignorance. It is very generally believed that previous to what is known as "quickening," there is no life, that that is the beginning, and any relief which may come before that period, is not a crime. I hold it to be one of the most sacred duties I have to perform to instruct my patients that life begins with the beginning of conception, and I find them in that regard very teachable and thankful. I have never yet found an intelligent woman, or even an ignorant woman, who was not glad to know that she had been saved from committing a crime. The idea of committing a murder had never occurred to her. I think the most of women who sin in this direction, sin through ignorance. I am sorry to say, our profession is largely at fault, because we have had all this moral and physiological teaching in our hands, and we have allowed women to grow up in our midst to marry and become the murderers of children, in ignorance of the real facts. The spirit of this article upholding infanticide, is a return to barbarism, which scientific medicine cannot for a moment tolerate.

